# Longitudinal dynamics and characterization of discriminative taxa in fecal microbiota of suckling and weaned piglets

**DOI:** 10.3389/fmicb.2026.1783640

**Published:** 2026-04-22

**Authors:** Bo Song, Shuo Yang, Boxuan Yang, Zhijian Xu, Feilong Deng, Ying Li

**Affiliations:** 1 Guangdong Provincial Key Laboratory of Animal Molecular Design and Precise Breeding, School of Animal Science and Technology, Foshan University, Foshan, China; 2 College of Animal Science, South China Agricultural University, Guangzhou, China; 3 Guangdong Laboratory for Lingnan Modern Agriculture, Guangzhou, China

**Keywords:** 16S rRNA sequencing, gut microbiota, microbial development, piglet, weaning

## Abstract

**Introduction:**

Early-life microbial colonization is fundamental to porcine health and production efficiency. However, the distinction between age-related development and weaning-induced shifts in the gut microbiota remains insufficiently characterized.

**Methods:**

This study investigated the longitudinal development of fecal microbiota in Duroc × Landrace × Yorkshire piglets from birth to day 28.

**Results and discussion:**

Using 16S rRNA gene sequencing, we first mapped the microbial development of suckling piglets, identifying a non-linear progression characterized by three distinct stages: initial colonization (days 1–7), rapid transition (days 14–21), and a stabilization stage (days 24–28). We then compared suckling piglets with those weaned at day 21. While no differences were observed on the day of weaning, weaned piglets exhibited significantly higher microbial richness and diversity by days 24 and 28 compared to suckling counterparts. β-diversity analysis confirmed a significant structural difference post-weaning, suggesting that weaning disrupt the developmental progress. Based on the LEfSe analysis and Random Forest models, *Lactobacillus* and *Collinsella* were identified as discriminative taxa in suckling and weaned piglets. These findings provide a map of microbial assembly and offer theoretical targets for mitigating weaning stress through microbial modulation.

## Introduction

The swine industry relies heavily on the healthy development of piglets during the early postnatal period to ensure long-term production efficiency. It is now widely accepted that the porcine intestinal microbiota plays a vital role in host health, contributing to immune system maturation, intestinal barrier integrity, and the transition of metabolic functions (Pluske et al., 2018). This microbial ecosystem is not a static entity but a dynamic community that undergoes rapid developmental changes from birth until several weeks post-weaning (Mach et al., 2015). Establishing a stable and diverse microbial community early in life is essential for protecting the host against enteric pathogens and optimizing nutrient absorption (Fouhse et al., 2016).

In intensive swine production, weaning is one of the most challenging periods in life of piglets. This transition, typically occurring around 21 days of age, involves an abrupt move from a liquid, highly digestible milk-based diet to a solid, plant-based diet (Campbell et al., 2013). This nutritional shift is exacerbated by environmental and social stressors, which frequently lead to weaning stress syndrome. This condition is characterized by intestinal dysbiosis, reduced growth performance, and increased susceptibility to post-weaning diarrhea (Gresse et al., 2017). Microbiologically, weaning often induces a significant reduction in the abundance of beneficial bacteria, such as *Lactobacillus*, and an increase in opportunistic pathogens like *Escherichia coli* (Lallès et al., 2007).

While extensive research has documented the impact of weaning on the porcine gut, most studies focus on the comparison between pre-weaning and post-weaning period. There is a lack of longitudinal research that compares weaning-induced changes against the natural microbial development that occurs in piglets that continue to suckle (Kim and Isaacson, 2015). Without a suckling control group beyond the weaning age, it is difficult to distinguish between changes caused by age-related development and those specifically triggered by the weaning process. In this study, we employed high-throughput 16S rRNA gene sequencing to map the longitudinal development of the fecal microbiota in piglets from birth to day 28. Our objective was to compare the microbial development of piglets weaned at 21 days of age with those that remained with the sow. By integrating alpha and beta diversity analyses with advanced statistical modeling, we aimed to identify specific driver or marker genera that define the weaning transition. The results from this study offer a high-resolution view of microbial assembly and provide a theoretical basis for managing gut health in early-life piglets.

## Materials and methods

### Animals

Duroc × Landrace × Yorkshire crossbred piglets were raised under uniform conditions in Guangzhou, China, following standard farm management protocols. The study was conducted in two parts. In the first part, 36 suckling piglets were slaughtered at 1, 7, 14, 21, 24, and 28 days of age, respectively (*n* = 6). In the second part, a separate group of piglets was weaned on day 21 and slaughtered at 21, 24, and 28 days of age (corresponding to the day of weaning, and 3- and 7-days post-weaning; *n* = 6) to allow for comparison with the suckling group. To eliminate potential bias from weight and gender, piglets of similar body weights were selected from different pens, maintaining a 1:1 male-to-female ratio. Piglets were humanely euthanized to allow for the collection of internal tissues for a broader study on intestinal physiology. For the present study, fecal samples were collected directly from the rectum of each piglet to avoid environmental contamination. Samples were immediately frozen in liquid nitrogen and stored at −80 °C. While fecal microbiota serves as a valuable proxy, it may not fully capture the distinct developmental patterns occurring in the small intestine. For details on the changes in the composition and function of the ileal microbiota, please refer to our previously published paper (Yang et al., 2025).

### 16 S rRNA sequencing

Total fecal DNA was extracted using the MolPure^®^ Stool DNA Kit (Yeasen, China) following the manufacturer’s instructions. DNA quality was assessed via 1% agarose gel electrophoresis and NanoDrop 2000. The V3–V4 region of the 16S rRNA gene was amplified using primers 341F (5’-ACTCCTACGGGAGGCAGCA-3’) and 805R (5’-GGACTACHVGGGTWTCTAAT-3’). Sequencing was performed on the Illumina NovaSeq 6000 platform with a paired-end read length of 250 bp.

### Bioinformatics

Raw reads were processed using the DADA2 pipeline in QIIME 2 to infer amplicon sequence variants (ASVs). During the denoising step, sequences with a quality score below 30 were filtered out (Yang et al., 2025). To normalize sequencing depth across samples, the feature table was rarefied to the minimum library size after filtration. Relative abundance was then calculated as the proportion of reads assigned to a specific taxon out of the total reads per sample. For visualization, taxa with a mean relative abundance ranking beyond the top 10 at the phylum level, or beyond the top 20 at the genus level, were consolidated into the “Other” category.

### Statistics

The analyses and data visualization were all performed using Graphpad 9.0 and MicrobiomeAnalyst at https://www.microbiomeanalyst.ca/ according to the guideline (Lu et al., 2023; Chong et al., 2020). For alpha diversity, Kruskal-Wallis tests were used for multi-group comparisons across ages, while Mann-Whitney U tests were used for pairwise comparisons between suckling and weaned groups. Beta diversity was analyzed via PERMANOVA (Permutational Multivariate Analysis of Variance, Bray-Curtis distance), and pairwise PERMANOVA was performed to identify differences between specific time points. LEfSe (Linear discriminant analysis-Effect Size) was performed at the genus level. Top 15 genera with Benjamini-Hochberg FDR-adjusted *P*-value < 0.05 and an LDA score > 2.0 are reported. Random Forest classifications were conducted at the genus level with parameters set as: 5000 trees to grow, seven predictors to try, and randomness turned on. Mean Decrease in Accuracy was used to rank the importance of genera. Correlation networks were constructed using the Spearman algorithm on the genus-level ASV table. Prior to analysis, data were centered log-ratio transformed. Only correlations with a coefficient (*r*) > 0.6 and *P* < 0.05 were visualized in the network. All specific statistical results are shown in the Supplementary materials.

## Results

### Changes in microbial diversity and overall composition of suckling piglets

**FIGURE 1:**
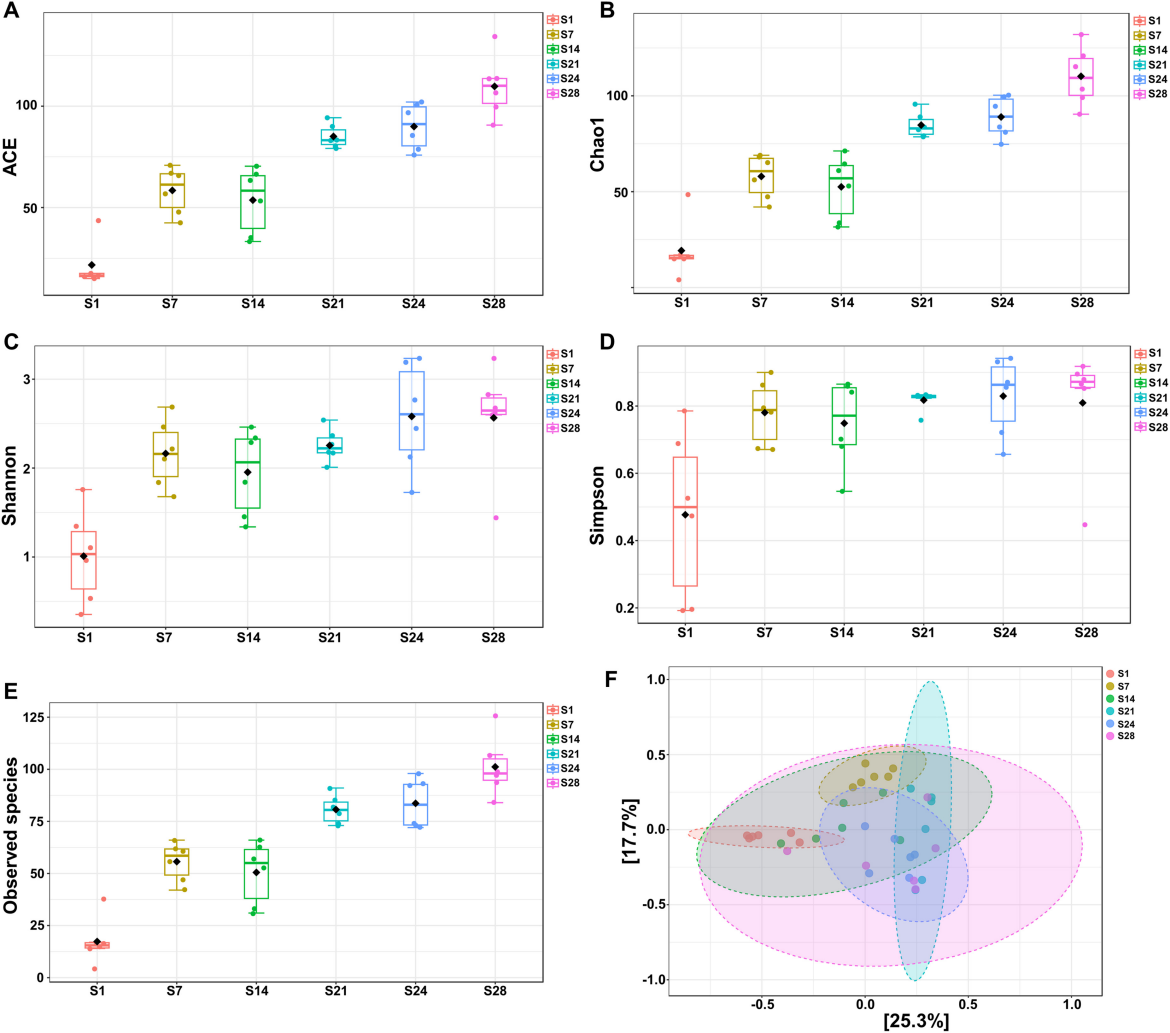
α- and β- diversity of fecal microbiota of suckling piglets at 1, 7, 14, 21, 24, and 28 days of age (*n* = 6). **(A–E)** ACE, Chao1, Shannon, Simpson, and Observed species indices. **(F)** Principal Coordinates Analysis (PCoA) plot of suckling piglets. S, suckling. Data are presented as Mean ± SEM. Detailed results of pairwise comparison are shown in Supplementary materials. Statistical significance threshold is *P* < 0.05.

From birth to 28 days of age, the α-diversity of fecal microbiota in piglets exhibited a distinct increasing trend with age (Figures 1A–E). Pairwise comparisons indicated (Supplementary Table 1) that the alpha diversity—including ACE, Chao1, Shannon, Simpson, and Observed species— of fecal microbiota at 1 day of age was significantly lower than that at 7, 14, 21, 24, and 28 days of age (*P* < 0.05). However, there was no significant difference between 7 and 14 days of age, nor between 21 and 24 days of age. Additionally, in pairwise comparisons across 7, 14, 21, 24, and 28 days of age, significant differences were observed in the ACE, Chao1, and Observed species indices (*P* < 0.05). However, no significant differences were found in the Shannon and Simpson indices. This suggests that while richness and diversity increased with age, evenness did not change significantly. The PCoA (Principal Coordinates Analysis) plot (Figure 1F) and pairwise comparisons (Supplementary Table 2) demonstrated significant separation among 1, 7, 14, 21, and 24 days of age, whereas no significant difference between 21 and 28 days of age, nor between 24 and 28 days of age. This indicates a substantial weekly variation before 24 days of age, followed by a stabilization of the microbiota composition thereafter.

### Microbial development patterns and identification of key genera in suckling piglets

**FIGURE 2:**
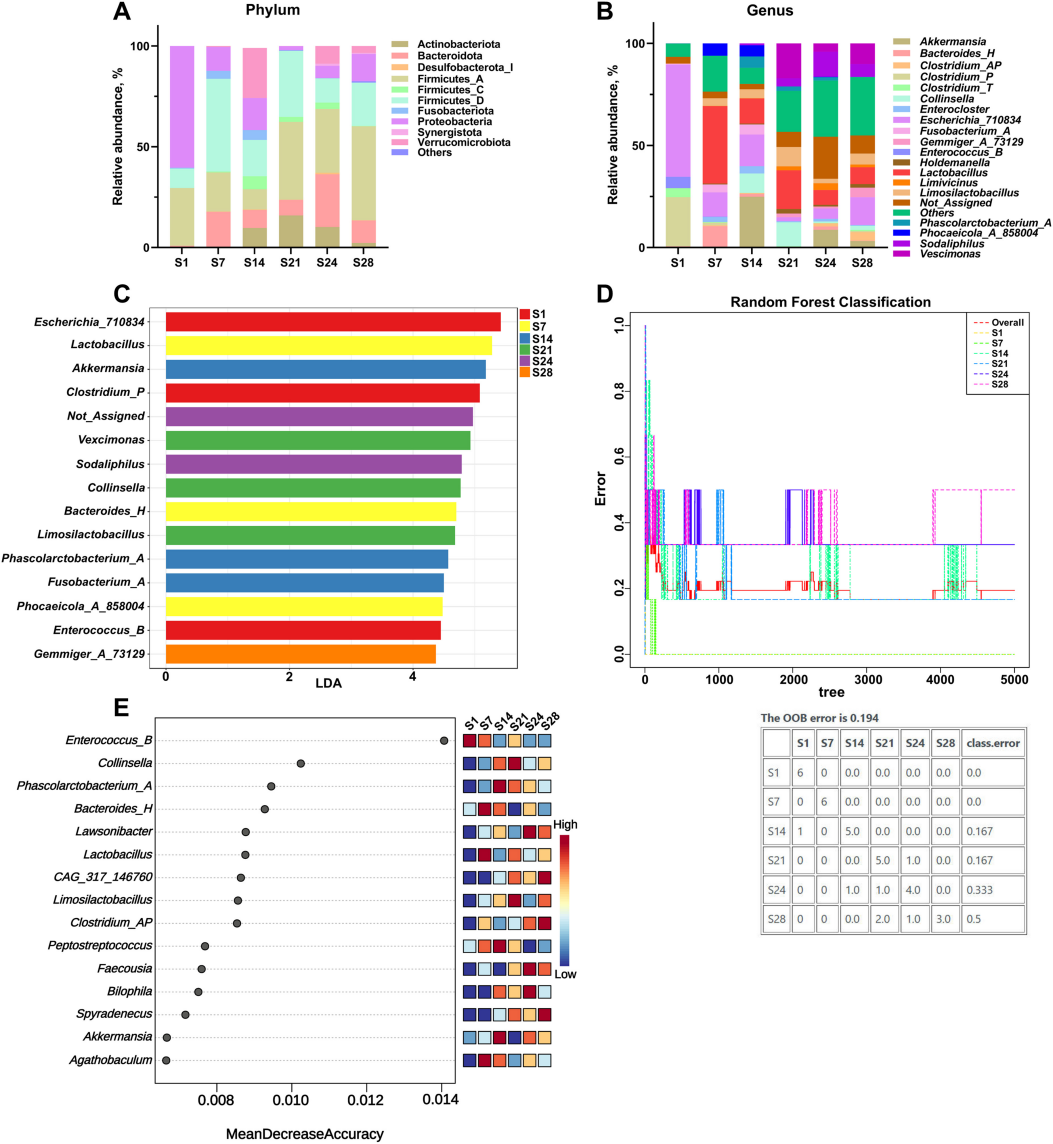
Analysis of the fecal microbial composition of suckling piglets at 1, 7, 14, 21, 24, and 28 days of age (*n* = 6). **(A)** Top 10 taxa at the phylum level. **(B)** Top 20 taxa at the genus level. **(C)** Key genera in LEfSe. **(D)** Random Forest classification model. **(E)** Key genera in Random Forest modeling. S, suckling. Detailed results of LEfSe are shown in Supplementary materials.

At the phylum level (Figure 2A), Firmicutes, Proteobacteria, and Bacteroidota are the dominant phyla in the feces of suckling piglets throughout 1–28 days of age. Verrucomicrobiota exhibited a transient, explosive increase at 14 days of age, followed by a rapid decline. Similarly, Actinobacteriota showed significant growth at 14 days of age, persisting until 24 days of age, before decreasing to lower levels by 28 days of age.

At the genus level (Figure 2B), *Escherichia_710834* and *Clostridium_P* were highly abundant in piglet feces at 1 day of age but rapidly disappeared or diminished thereafter. *Lactobacillus* and *Akkermansia* showed sudden increases and became dominant at 7 and 14 days of age, respectively. After 21 days of age, the abundance of *Collinsella* decreased significantly, while the abundance of other genera remained relatively uniform and stable.

LEfSe analysis (Figure 2C) identified the following biomarker genera for each age: 1 day of age: *Escherichia_710834*, *Clostridium_P*, and *Enterococcus_B*; 7 days of age: *Lactobacillus*, *Bacteroides_H*, and *Phocaeicola_A_858004*; 14 days of age: *Akkermansia*, *Phascolarctobacterium_A*, and *Fusobacterium_A*; 21 days of age: *Vescimonas*, *Collinsella*, and *Limosilactobacillus*; 24 days of age: *Sodaliphilus*; 28 days of age: *Gemmiger_A_73129*. See Supplementary Table 3 for detailed results of LEfSe analysis.

To identify the key drivers underlying the age-dependent development of gut microbiota in suckling piglets, a Random Forest classification model was established using age as the dependent variable. The model demonstrated robust predictive performance with an OOB (out-of-bag) error of 0.194 (Figure 2D), suggesting that microbial community structure effectively reflects the physiological age of the host.

Feature importance analysis highlighted the top 15 genera contributing most to age prediction (Figure 2E). Notably, a dendrogram constructed based on these key drivers (Figure 2D) clearly illustrated the phasic nature of microbial community development. Specifically, samples from piglets at 1 and 7 days of age clustered closely; samples from piglets at 14 and 21 days of age overlapped and clustered with the overall distribution; whereas samples from piglets at 14 and 21 days of age formed a distinct cluster. This pattern indicates that gut microbiota development in suckling piglets follows a progression characterized by three distinct developmental stages: (1) an initial colonization phase (days 1–7), (2) a rapid transition phase (days 14–21), and (3) a relatively stable phase (days 24–28).

### Comparison of fecal microbial diversity and composition between suckling and weaned piglets

**FIGURE 3:**
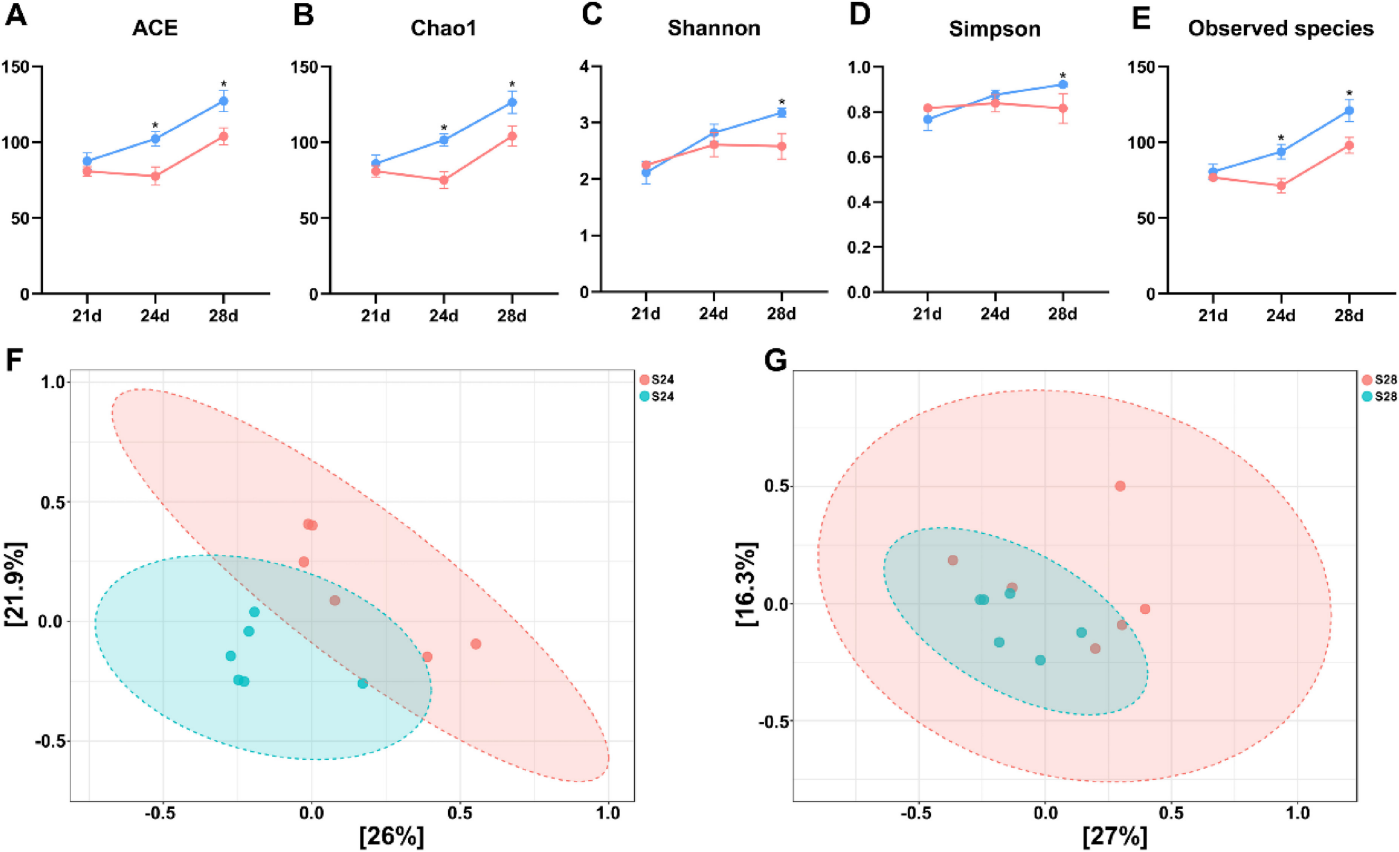
Comparison of fecal microbial α- and β-diversity between suckling and weaned piglets at 21, 24, and 28 days of age (*n* = 6). **(A–E)** ACE, Chao1, Shannon, Simpson, and Observed species indices. **(F)** Principal Coordinates Analysis (PCoA) plot of suckling and weaned piglets at 24 days of age. **(G)** PCoA plot of suckling and weaned piglets at 28 days of age. S, suckling; W, weaned. Data are presented as Mean ± SEM. Detailed results of pairwise comparison are shown in Supplementary materials. Statistical significance threshold is *P* < 0.05. *P < 0.05.

At 21 days of age, there were no significant differences in alpha diversity (Figures 3A–E) or beta diversity (Supplementary Figure 1A) between weaned and suckling piglets. The results of pairwise comparisons were shown in Supplementary Tables 4, 5. However, at 24 days of age, the ACE, Chao1, and Observed species indices in weaned piglets were significantly higher than those in suckling piglets (*P* < 0.05, Figures 3A,B,E). At 28 days of age, all the α-diversity indices in weaned piglets were significantly higher compared to the suckling group (*P* < 0.05, Figures 3C,D). Additionally, significant differences in beta diversity were observed between the two groups at both 24 and 28 days of age (*P* < 0.05, Figures 3F,G).

**FIGURE 4:**
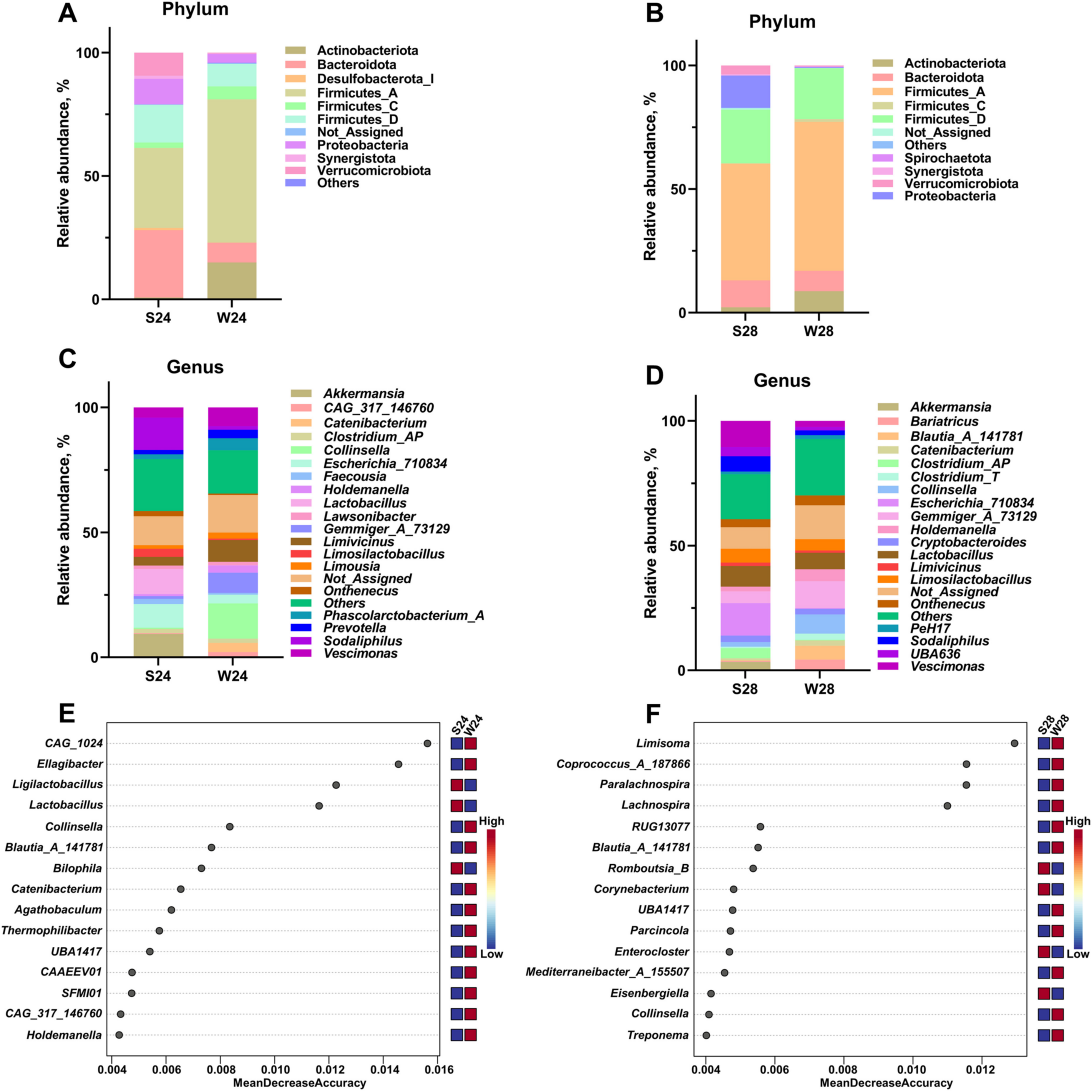
Comparison of fecal microbial composition between suckling and weaned piglets at 24 and 28 days of age (*n* = 6). Top 10 taxa at the phylum level in suckling and weaned piglets at **(A)** 24 and **(B)** 28 days of age. Top 20 taxa at the genus level in suckling and weaned piglets at **(C)** 24 and **(D)** 28 days of age. Key genera in Random Forest modeling of suckling and weaned piglets at **(E)** 24 and **(F)** 28 days of age. S, suckling; W, weaned.

At 21 days of age, the dominant phyla in the feces of both groups were Firmicutes, Bacteroidota, and Actinobacteriota (Supplementary Figure 1B). Interestingly, the phylum-level composition appeared more stable after weaning: Firmicutes, Bacteroidota, and Actinobacteriota remained the major phyla in weaned piglets at 24 and 28 days of age (Figures 4A,B). In contrast, in suckling piglets, Actinobacteriota was replaced by Verrucomicrobiota and Proteobacteria at 24 and 28 days of age, respectively (Figures 4A,B).

At the genus level, the abundance of *Collinsella* remained consistently high in weaned piglets (Figures 4C,D and Supplementary Figure 1C). *Lactobacillus* nearly disappeared 3 days post-weaning but showed partial recovery 7 days post-weaning, whereas its abundance remained consistently high in suckling piglets (Figures 4C,D and Supplementary Figure 1C). Since there were no differences in the alpha diversity and compositional structure of fecal microbiota between weaned and suckling piglets at 21 days of age, we only used the Random Forest model to analyze the fecal microbiota of weaned and suckling piglets at 24 and 28 days of age. The results showed that at both 24 and 28 days of age, *Collinsella*, *Blautia_A_141781*, and *UBA1417* were among the top 15 genera contributing to the differentiation between weaned and suckling piglet fecal microbiota as identified by the Random Forest model (Figures 4E,F).

### Analysis of discriminative taxa in suckling and weaned piglets

**FIGURE 5:**
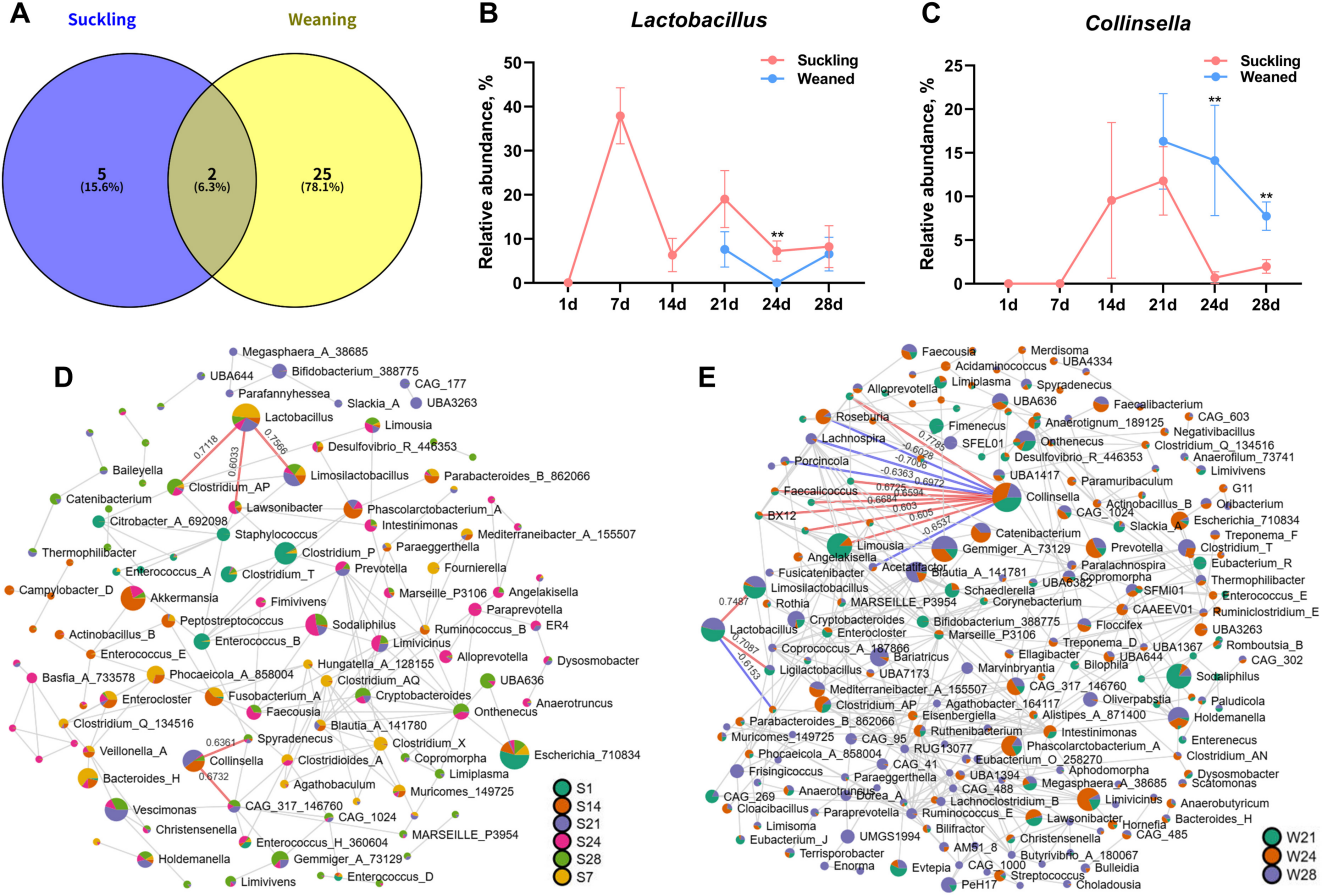
Analysis of discriminative taxa and correlation network (*n* = 6). **(A)** Intersection of key genera in suckling piglets at different ages and key genera affected by weaning. **(B)** Abundance changes of *Lactobacillus* in suckling and weaned piglets. **(C)** Abundance changes of *Collinsella* in suckling and weaned piglets. **(D)** Correlation network at the genus level of suckling piglets at 1, 7, 14, 21, 24, and 28 days of age. **(E)** Correlation network at the genus level of weaned piglets at 21, 24, and 28 days of age. S, suckling; W, weaned. Data are presented as Mean ± SEM. Detailed results of pairwise comparison are shown in Supplementary materials. Statistical significance threshold is *P* < 0.05. **P < 0.01.

Based on the results of LEfSe analysis and Random Forest models on suckling piglet feces, seven key genera were identified as potentially indicative of microbial development during the suckling phase. In the comparative analysis between weaned and suckling piglets, Random Forest model screened 27 key genera likely influenced by weaning. The intersection of these two sets yielded two genera—*Lactobacillus* and *Collinsella*—suggesting that they may be discriminative taxa in suckling and weaned piglets (Figure 5A). Abundance dynamics indicated that *Lactobacillus* peaked at 7 days of age and maintained a proportion of 10%–20% in suckling piglets; however, weaning caused an initial decline in its abundance followed by a recovery (*P* < 0.05, Figure 5B). In suckling piglets, *Collinsella* decreased rapidly after 21 days of age, whereas it remained at high levels in weaned piglets (*P* < 0.05, Figure 5C).

Species-level analysis revealed that the dominant *Lactobacillus* species in both suckling and weaned piglets were *Lactobacillus johnsonii* and *Lactobacillus delbrueckii*; however, their proportions within the total *Lactobacillus* population were reduced in weaned piglets (Supplementary Figures 1D,E). For *Collinsella*, *Collinsella vaginalis* and *Collinsella phocaeensis* were identified in suckling piglets, while weaned piglets additionally harbored *Collinsella bouchesdurhonensis*, though all were present in very low proportions (Supplementary Figures 1F,G). In general, the species-level information provided by 16S sequencing is limited and remains to be further elucidated by future metagenomic studies. Genus-level correlation networks (Figures 5D,E) revealed a simpler network structure in suckling piglets compared to a more complex one in weaned piglets, which is consistent with the finding of higher microbial diversity in weaned piglets.

## Discussion

The establishment of the intestinal microbiota in early life is a complex process that profoundly influences the maturation of the porcine immune system, gut barrier function, and nutrient absorption (Guevarra et al., 2019). In modern swine production, weaning represents the most severe stressor, frequently triggering growth retardation, intestinal inflammation, and heightened susceptibility to enteric pathogens (Tang et al., 2022). In this study, we utilized high-throughput 16S rRNA gene sequencing to map the longitudinal development of the fecal microbiota from birth to 28 days of age. Furthermore, we conducted a comparative analysis between continuously suckled piglets and those weaned at 21 days to differentiate the specific impacts of weaning stress from age-dependent microbial development. Our findings reveal a highly structured progression of microbial community assembly comprising three distinct developmental stages. Importantly, we identified *Lactobacillus* and *Collinsella* as discriminative taxa in suckling and weaned piglets.

Our data indicated that α-diversity increased significantly with age, a trend consistent with the ecological theory of development wherein an ecosystem progressively accumulates species to fill available niches (Sprockett et al., 2018). Random Forest and β-diversity analyses revealed that this microbial accumulation occurs in discrete phases. The initial colonization stage (days 1–7) was characterized by low diversity and the dominance of facultative anaerobes, notably *Escherichia_710834* and *Enterococcus*. This observation supports the oxygen-scavenging hypothesis of neonatal gut ecology (Gresse et al., 2017). At birth, the infant gut contains trace amounts of oxygen, which inhibits the growth of obligate anaerobes. Facultative anaerobes like *Escherichia* and *Enterococcus* act as pioneer species and rapidly consume the available oxygen, thereby remodeling the gut environment to make it suitable for the subsequent colonization of obligate anaerobes (Milani et al., 2017). Consequently, the high abundance of *Escherichia* at day 1 should be viewed not as pathogenic, but as a normal physiological state in the neonatal piglet. By day 7, the microbiota predominantly shifted toward members of Bacteroidetes and Firmicutes, particularly *Lactobacillus*. This shift reflects an adaptation to the primary available substrate: sow milk. *Lactobacillus* species are highly adapted to ferment lactose and milk oligosaccharides into lactate (Gänzle, 2015), creating an acidic environment that serves as a fundamental defense mechanism against pH-sensitive pathogens, such as *Salmonella* and enterotoxigenic *E. coli* (Fouhse et al., 2016).

A key finding of our study was the identification of a rapid transition stage (days 14–21), distinguished by a sharp increase in microbial complexity and the emergence of marker genus. Notably, *Akkermansia* (predominantly *A. muciniphila*) became a signature taxon around day 14. As a specialized mucin-degrading bacterium residing in the intestinal mucus layer (Belzer and de Vos, 2012), its proliferation at this age likely corresponds with the maturation of the piglet’s goblet cells and enhanced mucus secretion (Derrien et al., 2017). Widely considered a sentinel of gut health, *Akkermansia* degrades mucin to release free oligosaccharides and amino acids. These metabolites fuel a cross-feeding network that nourishes other beneficial bacteria, such as butyrate producers (e.g., *Faecalibacterium*), which cannot degrade mucin independently (Belzer and de Vos, 2012). This metabolic cross-talk is essential for energy harvest and signals the transition from a simple, milk-driven community to a complex, interdependent ecosystem. After the initial colonization stage (days 1–7) and the rapid transition stage (days 14–21), the gut microbiota of suckling piglets become relatively stable and more diverse. Our observation of a three-stage microbial development aligns with the previous meta-analysis (Dong et al., 2023), which highlighted predictable dynamic phases in commercial pigs.

Comparing weaned piglets with their age-matched, suckling counterparts revealed that weaning induced significantly higher α-diversity at 24 and 28 days of age. While elevated diversity is traditionally viewed as a hallmark of microbiota stability (Lozupone et al., 2012), this finding requires careful interpretation in the immediate post-weaning context. Suckling piglets maintained a high abundance of *Lactobacillus* due to the persistent and highly selective pressure of milk oligosaccharides, which enrich a narrow range of bacteria (Frese et al., 2015). The sudden withdrawal of milk, coupled with the introduction of plant-based solid feed, abruptly removes this selective pressure. This dietary shift likely breaks the *Lactobacillus* dominance, facilitating a sudden bloom of diverse environmental bacteria and solid-feed fermenters (Mach et al., 2015). Therefore, the observed increase in α-diversity post-weaning should not be directly equated with improved gut health; rather, it represents a transition from a stable, milk-organized community to a more heterogeneous state with lower functional resilience during the initial post-weaning period.

β-diversity analysis corroborated this shift, highlighting a prominent structural divergence at 24 and 28 days of age that indicates an abrupt reset of the microbial community. Based on LEfSe results and Random Forest modeling, we identified *Lactobacillus* and *Collinsella* as the discriminative genera. The most immediate and consistent consequence of weaning was the drastic depletion of *Lactobacillus* (Guevarra et al., 2019). This loss is well-documented to correlate with elevated intestinal pH and the potential overgrowth of enterotoxigenic *E. coli*, a primary etiological agent of post-weaning diarrhea (Lallès et al., 2007). In the present study, the dominance of *Lactobacillus* in the early suckling phase followed by a decrease post-weaning is consistent with the findings of previous studies (Wang et al., 2019; Luo et al., 2022). Our data underscore the necessity of targeted dietary interventions—such as acidified water or probiotics—between days 21 and 24 to compensate for this loss of *Lactobacillus* functionality and mitigate the critical post-weaning gap.

Additionally, *Collinsella*, a member of the phylum Actinobacteria, emerged as a discriminative marker. *Collinsella* species are implicated in bile acid metabolism and lipid absorption; in human and rodent models, their abundance correlates with serum cholesterol levels and systemic lipid metabolism (Astbury et al., 2019). For piglets, the dietary shift from highly digestible medium-chain triglycerides in milk fat to plant lipids in solid feed inevitably alters the intestinal bile acid pool. Consequently, fluctuations in *Collinsella* abundance may reflect the host’s physiological drive to modulate bile acid composition to efficiently digest the complex fats found in creep feed.

In summary, our study characterizes the development of the piglet gut microbiota as a three-stage process: an initial pioneer stage driven by facultative anaerobes, a transitional stage marked by the rise of *Akkermansia* and *Lactobacillus*, and a final stable stage. We demonstrate that weaning acts as a profound disruptor of this natural development, causing a diversity paradox where taxonomic richness temporarily increases due to the loss of milk-driven microbial dominance. Identifying *Lactobacillus* and *Collinsella* as discriminative taxa in suckling and weaned piglets provides novel targets for nutritional modulation. By specifically supporting these populations during the transition to solid feed, we can facilitate smoother physiological adaptations, thereby enhancing piglet survivability and growth performance. Future studies should integrate metagenomics, single-cell transcriptomics, and culturomics as demonstrated in recent microbiome research (Deng et al., 2026), to achieve species-level resolution of gut microbiota dynamics in piglets.

## Data Availability

The raw data supporting this study is available in the NCBI repository, BioProject: PRJNA1393927.

## Supplementary material

Supplementary Figure 1 Comparison of suckling and weaned piglets at 21 days of age, and species-level identification of discriminative taxa. **(A)** Principal Coordinates Analysis (PCoA) plot of suckling and weaned piglets at 21 days of age. **(B)** Top 10 taxa at the phylum level in suckling and weaned piglets at 21 days of age. **(C)** Top 20 taxa at the genus level in suckling and weaned piglets at 21 days of age. Species-level identification of **(D)** *Lactobacillus* and **(F)** *Collinsella* in fecal samples of suckling piglets. Species-level identification of **(E)** *Lactobacillus* and **(G)** *Collinsellain* fecal samples of weaned piglets. S, suckling; W, weaned. Detailed results of pairwise comparison are shown in Supplementary materials. Statistical significance threshold is *P* < 0.05.
