# Supplementary figures and images for: Longitudinal dynamics and characterization of discriminative taxa in fecal microbiota of suckling and weaned piglets
Source: Front Microbiol. 2026 Apr 22;17:1783640. doi: 10.3389/fmicb.2026.1783640 (PMC13144040; doi:10.3389/fmicb.2026.1783640)

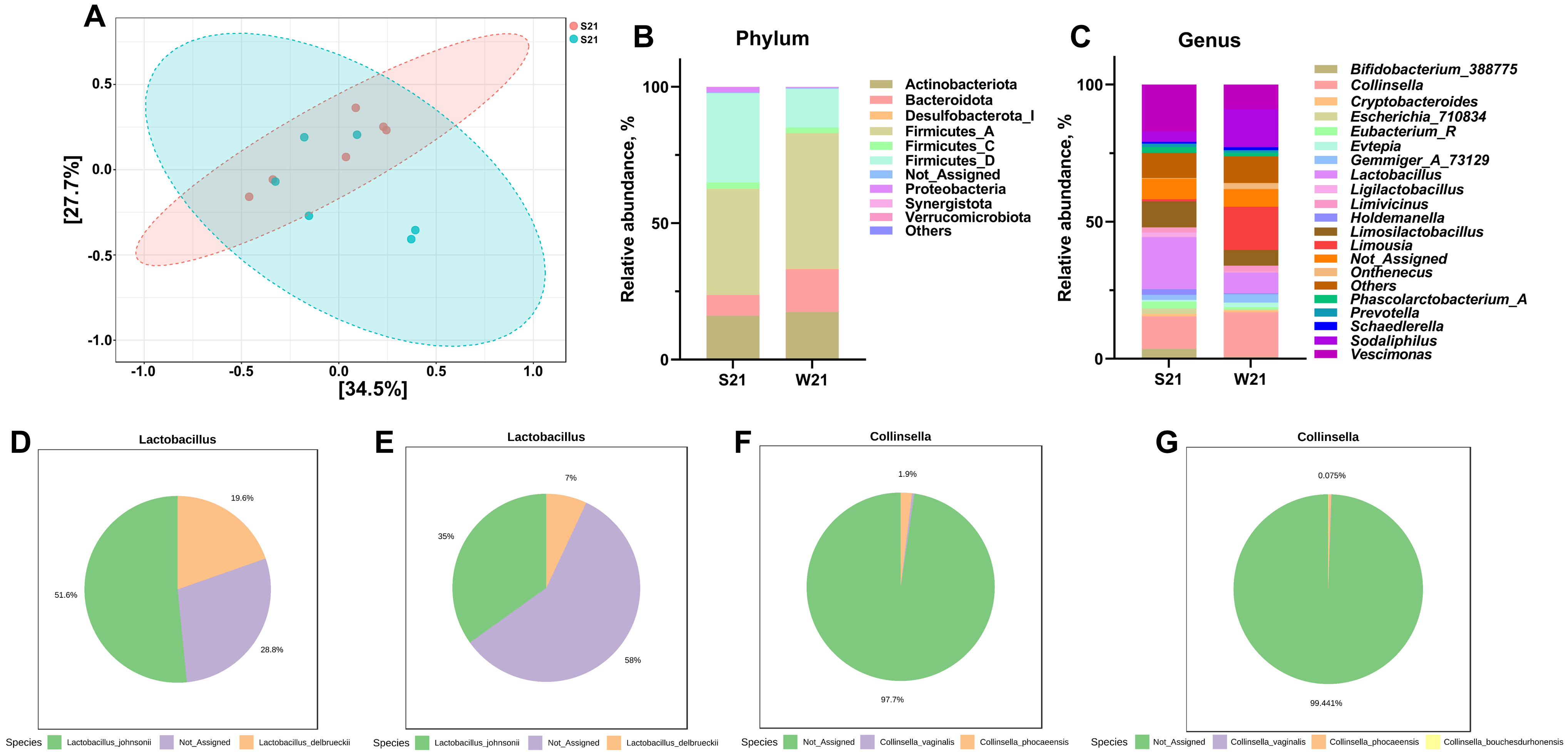

Supplement: Supplementary Figure 1 — Comparison of suckling and weaned piglets at 21 days of age, and species-level identification of discriminative taxa. (A) Principal Coordinates Analysis (PCoA) plot of suckling and weaned piglets at 21 days of age. (B) Top 10 taxa at the phylum level in suckling and weaned piglets at 21 days of age. (C) Top 20 taxa at the genus level in suckling and weaned piglets at 21 days of age. Species-level identification of (D) Lactobacillus and (F) Collinsella in fecal samples of suckling piglets. Species-level identification of (E) Lactobacillus and (G) Collinsellain fecal samples of weaned piglets. S, suckling; W, weaned. Detailed results of pairwise comparison are shown in Supplementary materials. Statistical significance threshold is P < 0.05. [file Image_1.tif]
